# Brief Research Report: Serum clara cell 16 kDa protein levels are increased in patients hospitalized for severe SARS-CoV-2 or sepsis infection

**DOI:** 10.3389/fimmu.2022.1037115

**Published:** 2022-10-13

**Authors:** Nathalie Rohmann, Paula Stürmer, Corinna Geisler, Kristina Schlicht, Katharina Hartmann, Kathrin Türk, Tim Hollstein, Florian Tran, Philip Rosenstiel, Andre Franke, Jan Heyckendorf, Stefan Schreiber, Dominik M. Schulte, Matthias Laudes

**Affiliations:** ^1^ Institute of Diabetes and Clinical Metabolic Research, University Medical Center Schleswig-Holstein, Kiel, Germany; ^2^ Division of Endocrinology, Diabetes and Clinical Nutrition, Department of Internal Medicine I, University Medical Center Schleswig-Holstein, Kiel, Germany; ^3^ Institute of Clinical Molecular Biology, Kiel University, Kiel, Germany; ^4^ Division of Pneumology, Department of Internal Medicine I, University Medical Center Schleswig-Holstein, Kiel, Germany

**Keywords:** CC16, SARS-CoV-2, sepsis, epithelial cell damage, biomarker

## Abstract

**Background:**

Clara cell 16 kDa protein (CC16) is a secretory protein primarily expressed in epithelial cells in the lungs. Previous studies show that CC16 exerts anti-inflammatory and immune-modulatory properties in both acute and chronic pulmonary diseases. However, despite the evidence of CC16’s high biomarker potential, evaluation of its role in infectious diseases is yet very limited.

**Methods:**

Serum CC16 concentrations were measured by ELISA and assessed in two different types of severe infections. Using a case-control study design, patients treated for either severe SARS-CoV-2 or severe non-pulmonary sepsis infection were compared to age- and sex-matched healthy human subjects.

**Results:**

Serum CC16 was significantly increased in both types of infection (SARS-CoV-2: 96.22 ± 129.01 ng/ml vs. healthy controls: 14.05 ± 7.48 ng/ml, p = 0.022; sepsis: 35.37 ± 28.10 ng/ml vs. healthy controls: 15.25 ± 7.51 ng/ml, p = 0.032) but there were no distinct differences between infections with and without pulmonary focus (p = 0.089). Furthermore, CC16 serum levels were positively correlated to disease duration and inversely to the platelet count in severe SARS-CoV-2 infection.

**Conclusions:**

Increased CC16 serum levels in both SARS-CoV-2 and sepsis reinforce the high potential as a biomarker for epithelial cell damage and bronchoalveolar−blood barrier leakage in pulmonary as well as non-pulmonary infectious diseases.

## Introduction

Infectious diseases are becoming increasingly important globally (1). As within the ongoing COVID-19 pandemic, novel pathogens (e.g. SARS-CoV-2) are causing a shift in risk factors responsible for severe disease progression. While in the past, unfavorable living conditions were the crucial determinant for severe infections, nowadays also obesity and type 2 diabetes with subsequent metabolic implications are becoming important as independent risk factors (2–4). Thus, the understanding of the underlying pathologies in the interaction of the immune system and metabolism is increasingly important.

Clara cell 16 kDa protein (CC16) is a secretory protein almost exclusively expressed in club cells (former clara cells) in the lungs and well detectable in circulation underlining its high biomarker potential. Consequently, CC16 has been comprehensively assessed in both acute [e.g. acute respiratory distress syndrome (ARDS)] and chronic [e.g. chronic obstructive pulmonary disease (COPD)] pulmonary conditions where it is described to exert anti-inflammatory and immune-modulatory properties (5). In addition, CC16 has been discussed as a marker for epithelial cell damage and bronchioalveolar leakage in situations of acute lung injury (6, 7). As one of the main interfaces to external pathogens, the respiratory system is extensively involved in the immune response, with infectious diseases often being focused within the lungs (8). However, even though numerous previous studies indicate CC16’s high biomarker potential in acute and chronic pulmonary implications (5), evaluation of its role in infectious diseases is yet very limited.

Given the high implication of SARS-CoV-2 infection in the lungs, with this study, we assessed serum CC16 in patients hospitalized for severe course of infection. To evaluate the disease specificity, we also included analysis of subjects affected by non-pulmonary sepsis that originates outside the lungs without primary respiratory infection.

## Patients and methods

### Study design and population

A case-control study design was used to assess whether circulating CC16 concentrations show alterations in n = 16 patients experiencing a severe course of SARS-CoV-2 infection and n = 12 patients experiencing a severe course of non-pulmonary sepsis infection compared to those of healthy control subjects. Patients have been treated at the intensive care unit of the University Medical Center Schleswig-Holstein (UKSH) in Kiel from 2013 to 2021. Here, data were collected as part of routine treatment procedure. Healthy control subjects were taken from the cross-sectional Food Chain Plus (FoCus) cohort and matched in a 1 case to 2 controls ratio to the nearest possible match using subject’s age and sex. As part of this population-based cohort, extensive subject data have been collected from 2011 to 2014, also comprising serum blood samples. Inclusion criterion for healthy control subjects was the availability of CC16 serum concentration, whereas exclusion occurred with the presence of either inflammatory, cardiovascular, metabolic or pulmonary chronic diseases. For the assessment of CC16’s disease specificity, comparison of SARS-CoV-2 and sepsis patients has been performed.

### CC16 measurement

CC16 in blood serum was measured by ELISA (ab238266, Abcam, Cambridge, MA, USA) according to manufacturer’s instructions. Samples were diluted 1:200, 1:250, 1:400 or 1:1000, depending on serum concentrations.

### Statistical analysis

Statistical analysis was carried out using R (9) and RStudio Version 1.2.5033 (RStudio., Inc., Boston, Massachusetts, USA). Continuous variables were tested for normal distribution with Shapiro-Wilk test. Because of small sample sizes, Student’s *t*-tests were applied for group comparisons. A subsequent in group association study has been performed using Spearman’s rank correlation test. For all analyses, statistical level was set at alpha <.05.

## Results

In the present study, we determined serum concentrations of CC16 in n = 16 subjects experiencing a severe SARS-CoV-2 infection and n = 12 subjects experiencing a severe sepsis infection who have been treated at the intensive care unit at UKSH in Kiel, Northern Germany. Basic characteristics of the study population are provided in **Table 1**. Due to the small sample sizes, we performed case-control comparisons using 1:2 age- and sex-matched healthy control subjects from our cross-sectional FoCus cohort, comprising n = 497 individuals with available CC16 serum concentrations. In total, n = 32 healthy controls were examined in case of SARS-CoV-2 and n = 24 healthy controls in case of sepsis of non-pulmonary focus.

**Table 1:** Characterization of the study population.

	SARS-CoV-2 infection			Non-pulmonary sepsis infection			p_INF_
	Cases	healthy controls	p_SARS_	cases	healthy controls	p_SEP_	
subjects, n (%)	16 (25.0)	32 (75.0)	–	12 (25.0)	24 (75.0)	–	–
female sex, n (%)^a^	5 (31.25)	16 (50.0)	0.35	7 (58.33)	16 (75.0)	0.90	0.30
age, years^b^	54.44 ± 19.93	55.5 (34.75; 61.0)	0.3	68.17 ± 12.44	58.08 ± 5.98	0.02	0.03
CRP, ng/ml^b^	77.02 ± 62.96	1.0 (0.9; 1.2)	2.25 x 10^-3^	224.2 (124.7; 279.2)	1.0 (0.9; 1.13)	7.61 x 10^-5^	3.04 x 10^-3^
IL-6, pg/ml^b^	90.21 ± 149.92	2.7 (2.15; 4.6)	0.05	–	3.55 (2.15; 4.9)	–	–
CC16, ng/ml^b^	96.22 ± 129.01	14.05 ± 7.48	0.022	35.37 ± 28.10	15.25 ± 7.51	0.032	0.09

Data was tested for normal distribution using Shapiro-wilk test. Normally distributed data are presented as mean ± standard deviation, non-normally distributed data as median (25th, 75th percentile). Categorical variables are presented as number of subjects (percentages). ^a^ Statistical significance was tested using Chi^2^-test. ^b^ Statistical significance was tested using t-tests. p_SARS_, comparison of SARS-CoV-2 cases and healthy controls; p_SEP_, comparison of sepsis cases and healthy controls; p_INF_, comparison of SARS-CoV-2 and sepsis cases. CRP, C-reactive protein; IL-6, interleukin 6; CC16, clara-cell 16 kDa protein.

### SARS-CoV-2

**Figure 1:**
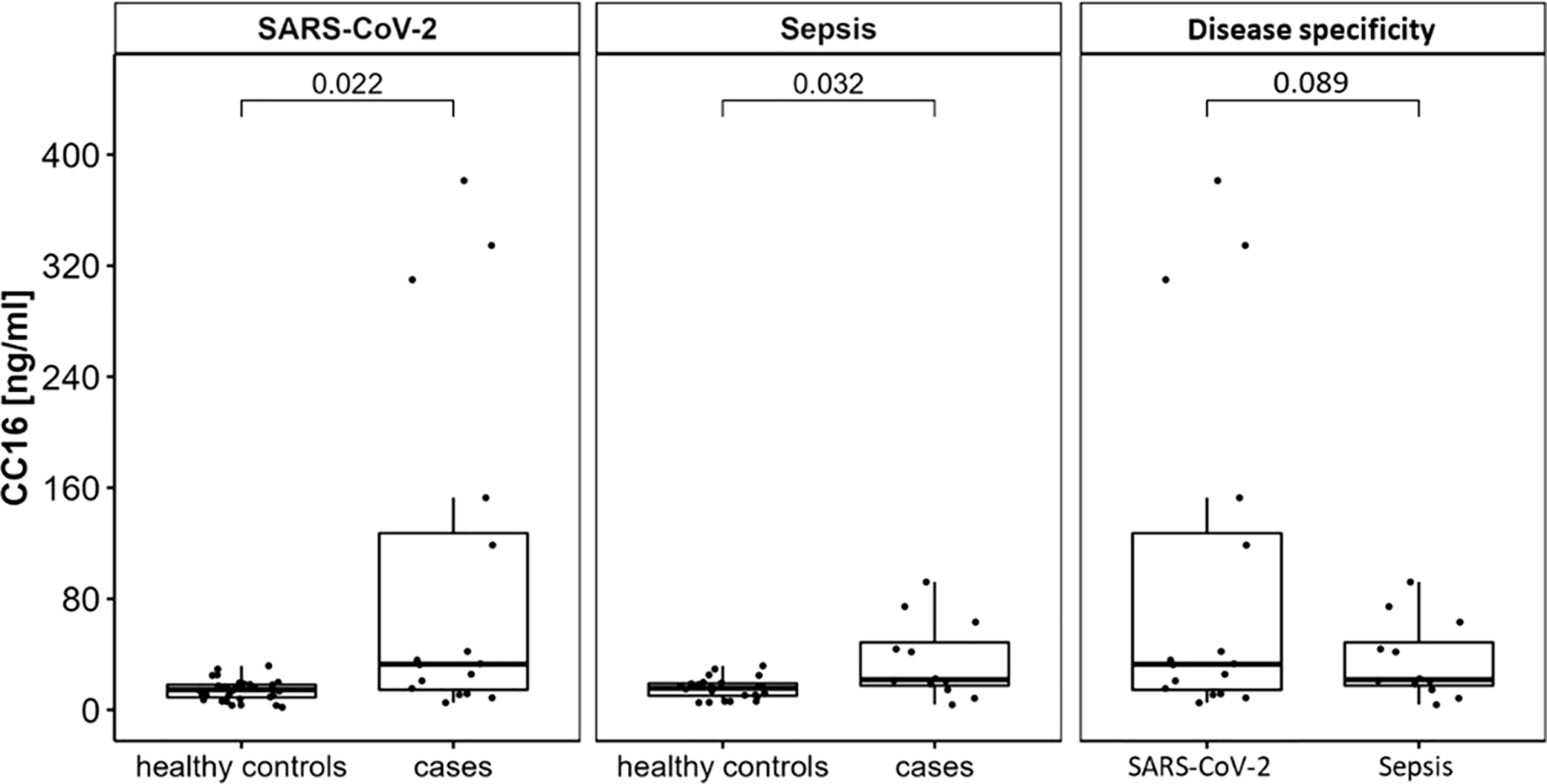
CC16 serum concentrations are significantly increased in patients experiencing either SARS-CoV-2 or non-pulmonary sepsis infection. Serum levels of n = 16 subjects hospitalized for severe SARS-CoV-2 infection and of n = 12 subjects hospitalized for severe sepsis infection were compared to age- and sex-matched healthy control subjects using Student’s t-tests. This displays significant higher serum concentrations in severe infection with and without pulmonary focus of infection. Data are presented as individual CC16 serum levels and as boxplots indicating the median, 25th, 75th percentile, minimum and maximum values.

14 of the 16 subjects encountering SARS-CoV-2 infection have recovered and been discharged from the hospital. The infection was fatal for two male patients, who have both previously been severely multimorbid, however they did not display any significant differences in any of the assessed variables compared to the survivors including CC16 serum levels. Most patients were reported to be within normal weight range, only 2 patients were overweight and 2 were obese. Type 2 diabetes was present in 5 (31.25 %), and arterial hypertension in 8 (50 %) of the patients. Assessed by *t*-test, patients with SARS-CoV-2 infection show significant higher CC16 serum levels compared to healthy control subjects (p = 0.02, see **Figure 1** left). When assessing individual CC16 serum levels, it shows that 3 of the 16 SARS-CoV-2 infected participants display significantly higher CC16 levels than the other 13 participants (p = 4.49 x 10^-4^) which is associated with higher participants’ age (p = 1.92 x 10^-2^). Furthermore, 2 of the 3 participants are reported to be comorbid with a heart disease. Additional correlation analysis of CC16 with clinical measures of disease progression within the SARS-CoV-2 infected participant group reveals a strong positive association with the disease duration (r_S_ = 0.797, p = 3.69 x 10^-4^) but not with the disease progression scale introduced by the World Health Organization (10). Correlation analysis further reveals a significant inverse association with the blood platelet counts (r_S_ = -0.625, p = 9.56 x 10^-3^) by assessing CC16 concentrations in relation to functional blood markers (see **Table 2**).

**Table 2:** Correlation analysis of CC16 with clinical measures and immunological serum markers in n = 16 patients treated for SARS-CoV-2 infection.

	SARS-CoV-2 infection	r_S_	p-value
subjects, n (%)	16 (100)	–	–
time since diagnosis, days	31 (15; 38)	0.797	3.69 x 10^-4^
WHO clinical progression scale, 0-10	7 (5; 7)	0.312	0.24
age, years	54.44 ± 19.93	0.423	0.10
CRP, ng/ml	77.02 ± 62.96	-0.024	0.93
IL-6, pg/ml	43.05 (19.55; 61.65)	0.196	0.48
D-dimer, ng/ml	2.85 (1.66; 5.83)	0.302	0.32
platelets, 10^9^/L	220 (126.5; 303.2)	-0.625	9.56 x 10^-3^
leucocytes, 10^9^/L	9.74 (6.14; 15.9)	-0.191	0.48
lymphocytes, 10^9^/L	1.1 (1.04; 1.15)	-0.300	0.68
granulocytes, 10^9^/L	7.12 (6.25; 14.9)	0.300	0.68
GOT, U/l	95.9 (54.95; 199.03)	-0.132	0.62
GGT, U/l	336.5 (172.8; 596.8)	-0.104	0.70
creatinine, µmol/l	110 (51.25; 157.5)	0.471	0.07

Data was tested for normal distribution using Shapiro-wilk test. Normally distributed data are presented as mean ± standard deviation, non-normally distributed data as median (25th, 75th percentile). Categorical variables are presented as number of subjects (percentages). Statistical significance was tested using Spearman’s rank correlation tests. CRP, C-reactive protein; IL-6, interleukin-6, GOT, glutamate oxaloacetate transaminase, GGT, gamma-glutamyltransferase.

### Sepsis of non-pulmonary focus

Out of the 12 patients treated for sepsis, the infection was fatal for one male patient. Infection was either located mainly in the gastrointestinal tract (n = 4, 33.33 %), bile duct (n = 2, 16.67 %), or kidneys (n = 1, 08.33 %), or remained unknown (n = 5, 41.57 %). Most patients were mildly obese with a mean BMI of 32.69 ± 10.13 kg/m^2^. In sepsis patients, higher CC16 levels were displayed in the case-control comparison with healthy control subjects (p = 0.032, see **Figure 1** middle).

### Comparison of SARS-CoV-2 and sepsis of non-pulmonary focus

Comparison of patients suffering from SARS-CoV-2 infection with patients suffering from sepsis with non-pulmonary focus reveals no distinct differences between the type of infection (p = 0.089, see **Figure 1** right).

## Discussion

As the ongoing COVID-19 pandemic emphasizes the global significance of infectious diseases, understanding disease mechanisms is mandatory. Clara cell 16 kDa protein (CC16), also known as uteroglobin, CC10 or secretoglobulin, is a protein mainly secreted by non-ciliated bronchial epithelial cells in the respiratory tract. Its easy detection in blood circulation offers high biomarker potential especially in pulmonary diseases (11). Consequently, CC16 has been extensively assessed in multiple chronic and acute lung conditions concluding properties for the evaluation of lung epithelial injury in e.g. COPD, asthma, idiopathic pulmonary fibrosis, ARDS, and sarcoidosis (5). However, despite the major role of the pulmonary system in the immunological defense of infections, evaluation of CC16 in infectious diseases is yet very limited.

With our finding of significantly increased CC16 concentrations in SARS-CoV-2 infection, we provide a contradictory result to a study from Yin et al. who demonstrated significantly decreased serum CC16 in n = 9 Chinese patients suffering from critical COVID-19 infection compared to n = 7 healthy control subjects. Here, significantly reduced CC16 mRNA in the lungs resulting from epithelial cell damage was proposed as a potential mechanical explanation (12). Decreased CC16 serum levels are rather characteristic in chronic lung diseases with high degrees of bronchial epithelial cell damage, as it was recently shown by Li et al. who demonstrated an association of CC16 mRNA expression levels and asthma susceptibility, severity and exacerbations (13).

On the contrary, our result is more consistent with previous studies of acute pulmonary damage concluding higher detection of CC16 in circulation due to an increased permeability and leakage of the bronchoalveolar-blood barrier (6, 14). We were additionally able to display an association of CC16 levels with the duration of infection, which is in line with a recent study of Tiezzi et al. who not only demonstrated a similar increase of serum CC16 but also a positive correlation with a fatal outcome in n = 32 ICU-treated SARS-CoV-2 patients (15). Moreover, previous studies displayed high CC16 serum concentrations in ARDS (16, 17) and pulmonary fibrosis (18, 19), both complications observed with severe COVID-19 infection (20).

Sibila et al. who assessed CC16 serum levels in relation to the lung diffusion capacity in Covid-19 survivors six months after hospital discharge did not show any significant differences between participants with normal or low “diffusing capacity of the lung for carbon monoxide” (DLCO) test. Still, the median CC16 levels of the assessed groups both exceed the median level seen in our healthy control subjects (21) which might suggest dysregulated CC16 passage from the lungs into the circulation even 6 months after the infection independent of DLCO.

We additionally found a significant inverse correlation of CC16 and platelets, which is very interesting in the context of epithelial cell damage, since higher platelet counts have previously been described to be protective in infection-induced lung injury in a mouse model (22). These data may allow the suggestion of a sequential influence on CC16 levels during SARS-CoV-2 infection, in which both the impairment of CC16 expression and increase of permeability of the bronchoalveolar-blood barrier could be dependent on the severity of disease course. Yet, for a precise conclusion, further studies, especially on a mechanistic level, are needed.

Acute lung injury (ALI) and ARDS can also be observed in the progression of non-pulmonary sepsis without primary signs of lung infection (23). Since, due to its small particle size, CC16 has been proposed as an indicator for even a very mild damage of the bronchoalveolar-blood barrier (7), high CC16 serum concentrations, as we observed in sepsis cases particularly chosen with other focus of infection, may be useful for the prediction of lung injuries in non-pulmonary sepsis.

Overall, significantly increased serum CC16 in both SARS-CoV-2 and non-pulmonary sepsis underline the huge biomarker potential of CC16 in infectious diseases as well as its independence of severe lung implication. To assess the actual extent of CC16’s potential for the prediction of disease course or even outcome, further comprehensive studies are needed.

## Data availability statement

The raw data supporting the conclusions of this article will be made available by the authors, without undue reservation.

## Ethics statement

The studies involving human participants were reviewed and approved by Ethics Committee of the Medical Faculty, Kiel University, Germany. The patients/participants provided their written informed consent to participate in this study.

## Author contributions

ML conceived the research idea. NR, PS, and KH performed laboratory measurements. NR performed main data analysis and wrote the manuscript with support from CG, KS, and KT, FT, PR, and SS provided SARS-CoV-2 patient data and aided with corresponding data processing. DS provided sepsis patient data and aided with corresponding data processing. TH, AF, JH, and ML were involved in the interpretation of results and contributed medical expertise of different areas. All authors contributed to the article and approved the submitted version.

## Conflict of interest

The authors declare that the research was conducted in the absence of any commercial or financial relationships that could be construed as a potential conflict of interest.

## Publisher’s note

All claims expressed in this article are solely those of the authors and do not necessarily represent those of their affiliated organizations, or those of the publisher, the editors and the reviewers. Any product that may be evaluated in this article, or claim that may be made by its manufacturer, is not guaranteed or endorsed by the publisher.
